# Repaglinide-Induced Acute Pancreatitis

**DOI:** 10.7759/cureus.16983

**Published:** 2021-08-07

**Authors:** Ndausung Udongwo, Anton Mararenko, Halah Alchalabi, Tasnuva Amin, Christopher Lesniak, Umar Sharif Khawaja

**Affiliations:** 1 Internal Medicine, Jersey Shore University Medical Center, Neptune, USA; 2 Endocrinology, Diabetes and Metabolism, Mount Sinai Medical Center, New York , USA

**Keywords:** repaglinide, acute pancreatitis, gallstones, alcohol, medication-induced pancreatitis

## Abstract

Acute pancreatitis is a common reason for hospitalization in the United States and can have a high degree of morbidity and mortality if not treated appropriately. Establishing the diagnosis and following guideline-directed medical therapy are both important. In the Western world, the most common causes include acute alcohol overuse, hypertriglyceridemia, gallstone pancreatitis, post-instrumentation including endoscopic cholangiopancreatography, and medication side effects. Our team describes the case of an 84-year-old male that was found to have acute pancreatitis secondary to repaglinide, a commonly used medication for the management of diabetes mellitus. The diagnosis was made based on the imaging findings, physical examination, and the corresponding laboratory markers. The patient was also found to have a blood-alcohol level at baseline and triglyceride levels within normal range. The patient’s symptoms resolved with the cessation of repaglinide administration. Our team hopes to make the medical community more aware of the potential association between repaglinide and the potentially rapidly debilitating disease.

## Introduction

Acute pancreatitis (AP) is caused by the aberrant activation of proteolytic enzymes within the pancreas. With more than 230,000 cases each year, it is one of the most common causes of hospitalization in the United States [1]. There are several etiologies of this debilitating disease that has already been reported in the medical literature. Cholelithiasis (40%-70%) and alcohol abuse (25%-35%) are still regarded as the most common causes of AP; other causes include trauma, infections, toxins, metabolic abnormalities (hypertriglyceridemia, hypercalcemia), autoimmune diseases, and medication-induced pancreatitis (MIP) [1,2]. On the other hand, MIP is rare (0.1%-2%) in the general population [1,2]. Repaglinide is a very rare culprit agent of AP. We report a case of an 84-year-old male with controlled type 2 diabetes who was admitted and treated for AP. Laboratory and imaging tests ruled out common causes of AP. Their symptoms improved after repaglinide was held and correlated with a progressively decreasing lipase level. Our case is rare, and to our knowledge, this is the second case reported in the medical literature of this medication side effect. 

## Case presentation

An 84-year old male with a past medical history of diabetes, hyperlipidemia, hypertension, hypothyroidism, gastroesophageal reflux disease, asthma, cholelithiasis status post cholecystectomy, and prostate cancer status post radiation therapy presented to the emergency department (ED) with complaints of worsening epigastric/left upper quadrant abdominal pain that started a day prior to admission. The pain was 8/10 in severity, non-radiating, and associated with nausea and non-bilious vomiting. He denied any fever, chills, recent trauma, weight loss, change in diet, or sick contacts. He never smoked tobacco and denied any recent alcohol consumption. Family history was unremarkable. Surgical history was remarkable for cholecystectomy. He was allergic to ciprofloxacin and penicillins (mild rash). His medication regimen included repaglinide 3 mg three times daily, amlodipine 5 mg daily, simvastatin 20 mg daily, metformin 1000 mg twice daily, levothyroxine 100 mg daily, montelukast 10 mg daily, and pantoprazole 40 mg daily. Upon presentation, his initial vitals showed a blood pressure of 151/79 mmHg, a heart rate of 79 beats per minute, a respiratory rate of 18 breaths per minute, a temperature of 98.1℉, and oxygen saturation of 98% on room air. Physical examination demonstrated epigastric/left upper quadrant tenderness without rebound, guarding, or organomegaly. Sclera and skin were anicteric, and cardiopulmonary examination was unremarkable. 

 

The laboratory results are shown in Table 1. 

**Table 1:** Summary of laboratory results on admission

Laboratory study	Results	References
Hemoglobin (g/dL)	12.2	12-17.5
White blood cell count (10^3^/uL)	8.1	4.5-11
Glucose (mg/dL)	238	70-110
Blood urea nitrogen (mg/dL)	30	5-25
Creatinine (mg/dL)	1.39	0.61-1.24
Amylase (U/L)	78	28-100
Lipase (U/L)	3010	20-55
Calcium (mg/dL)	9.4	8.5-10.5
Aspartate aminotransferase (U/L)	382	10-42
Alanine aminotransferase (U/L)	118	10-60
Triglyceride (mg/dL)	71	0-150
Hemoglobin A1c (%)	6.6	<5.7
Blood alcohol level (%)	0.000	<0.005
Total bilirubin (mg/dL)	3.5	0.2-1.3
Alkaline phosphatase (U/L)	45	38-156

**Figure 1:**
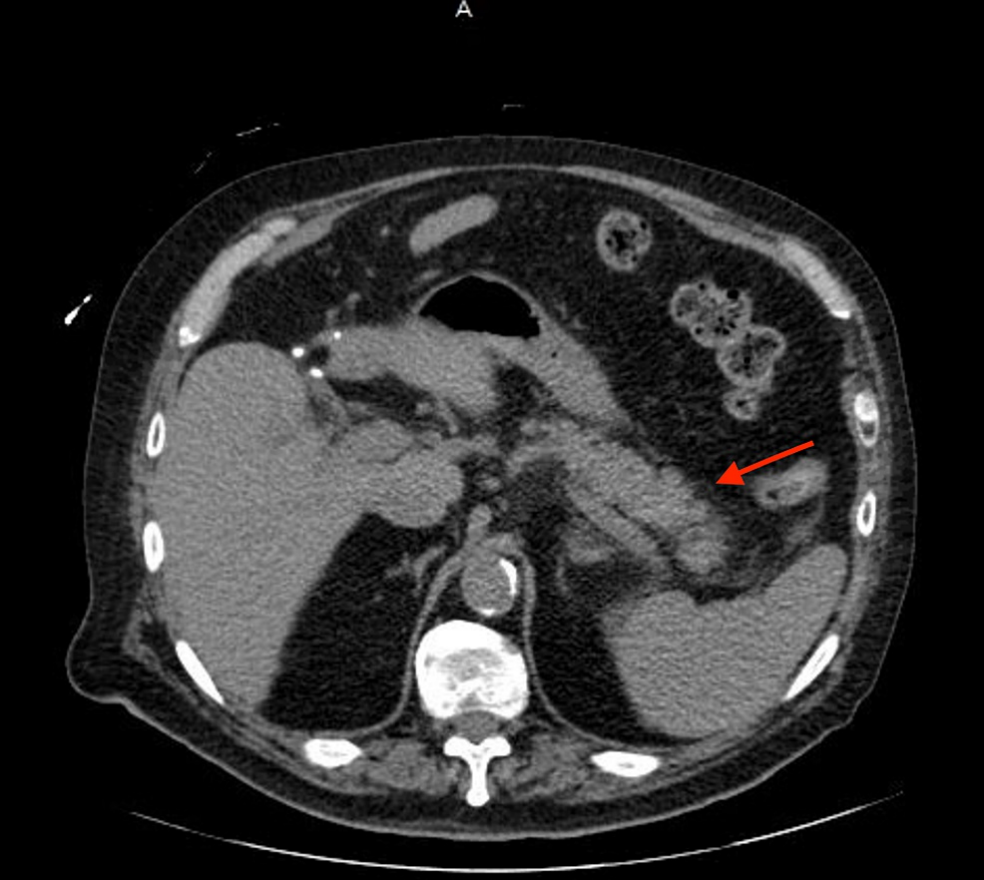
Computed tomography scan of the abdomen/pelvis demonstrating findings associated with acute pancreatitis (red arrow) including diffuse enlargement of the pancreas in addition to an irregular contour of the pancreatic margins, and peripancreatic fat stranding.

**Figure 2:**
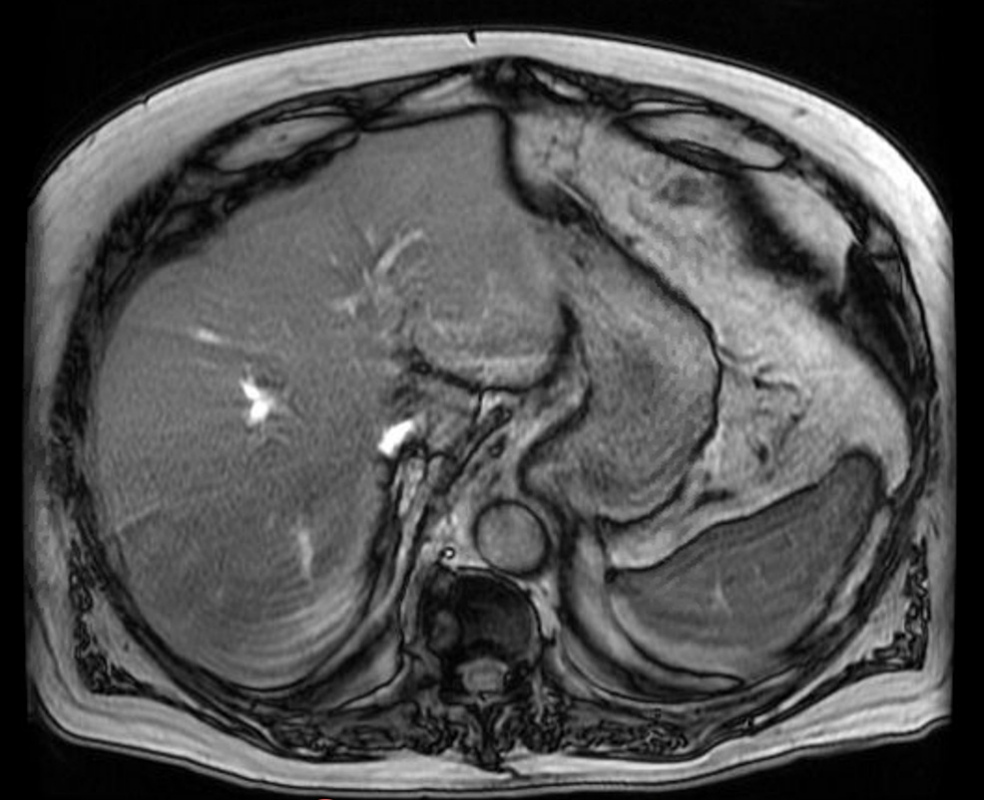
Magnetic resonance cholangiopancreatography scan showing absence of gallstones or biliary duct obstruction.

Computed tomography (CT) scan of the abdomen and pelvis revealed inflammatory changes surrounding the head of the pancreas (Figure 1). Right upper quadrant ultrasound showed mild hepatomegaly and absence of fluid collection in gallbladder fossa (post-cholecystectomy), with biliary duct dilation. He was diagnosed with AP. Oral intake was restricted and he was treated with intravenous fluids, morphine, and promethazine. His blood glucose was only controlled with an insulin sliding scale, while his oral hyperglycemic medications were held. Magnetic resonance cholangiopancreatography (MRCP) imaging confirmed findings consistent with AP with no significant post-cholecystectomy biliary ductal dilatation (Figure 2). On day 2 of admission, his symptoms had completely resolved. Also, his serum lipase level decreased to 555 U/L. Due to high suspicion for drug-induced pancreatitis, he was advised to stop taking the repaglinide. He was discharged home on metformin and scheduled for outpatient gastroenterology and endocrinology follow-up. On his two-week follow-up visit, his serum lipase was 80 U/L, and blood glucose was well controlled with metformin.

## Discussion

AP is one of the most common causes of hospitalization in the United States [1]. Several etiologies to this debilitating disease have already been reported in the medical literature. More than 525 medications have already been reported to induce AP [3]. It is rare with an incidence of about 0.1%-2% [1-4]. There should be a high index of suspicion when other common causes are ruled out, especially in the setting of a patient taking a medication with known side effects [3]. Repaglinide, on the other hand, is a “zebra amongst the horses.” Our patient had symptoms together with laboratory and imaging findings consistent with AP. Alcohol and gallstones-induced pancreatitis were ruled out from our workup. Blood sugar was controlled with sliding scale insulin while holding his oral hypoglycemic agents. His symptoms and lipase levels decreased over time. An increased risk of this disease may be higher in the older population, due to the high rate of polypharmacy [5]. Clearly, our patient fits well into this. Also, some studies have reported increased risk in pediatric and human immunodeficiency virus patients [5], with the latter reported to have an incidence of about 14% [4,5]. Usually, mild to moderate presentations are seen with MIP [1,5]. 

The pathophysiology of MIP is not clear. Direct medication toxicity, toxic metabolites released from various medications, and proteolytic activation of pancreatic enzymes caused by these medications are some of the proposed mechanisms [5]. Physiologically, inactive zymogens secreted by pancreatic cells are released and only activated in the duodenum. The pancreas also has a defense mechanism (e.g. trypsin inhibitor) present to halt any unusual enzymatic activation [6]. Aberrant activation of these cells (zymogens) while in the pancreas or disinhibition of the defense system may result in AP [1,6]. Angiotensin-converting enzyme inhibitors mechanism of pancreatitis is suggested to be likely due to its kallikrein-kinin system interaction, resulting in pancreatic duct angioedema [7]. Activating the withdrawal/cessation test in medications suspicious for MIP could have an immediate effect, and help in diagnosis, treatment, and prevention of AP [1]. 

There have been reports of medications used to control diabetes that can cause AP. Sulfonylureas and glucagon inhibitory peptide 1 agonists increase the risk for AP. On the other hand, insulin and metformin have been reported to decrease the risk of MIP [5]. Our patient was on metformin as well; however, he needed repaglinide for better glycemic control. CT scan of abdomen and MRCP ruled out post-cholecystectomy gallstones pancreatitis. His BAL was also within normal limits. After the withdrawal of repaglinide, he had immediate relief of symptoms. We recommend careful medication reconciliation in all patients presenting to the ED with AP, especially on the notorious medications causing this side effect. To our knowledge, this is the second case of repaglinide-induced AP that has been reported in the medical literature [5]. 

## Conclusions

There should be a high index of suspicion with patients presenting with AP who are on any medication associated with this disease. Repaglinide is a drug rarely associated with AP and has only been reported once in the medical literature. Early cessation of such medication may reduce complications and decrease mortality. We recommend that more cases be reported on medications associated with similar side effects. 
